# Adiponectin Improves In Vitro Development of Cloned Porcine Embryos by Reducing Endoplasmic Reticulum Stress and Apoptosis

**DOI:** 10.3390/ani11020473

**Published:** 2021-02-10

**Authors:** Muhammad Rosyid Ridlo, Eui Hyun Kim, Anukul Taweechaipaisankul, Byeong Chun Lee, Geon A. Kim

**Affiliations:** 1 Department of Theriogenology and Biotechnology, Research Institute for Veterinary Science, College of Veterinary Medicine, Seoul National University, Seoul 08826, Korea; rosyidridlodrh@gmail.com (M.R.R.); hyun9214@snu.ac.kr (E.H.K.); famfamat@gmail.com (A.T.); bclee@snu.ac.kr (B.C.L.); 2 Department of Bioresources Technology and Veterinary, Vocational College, Universitas Gadjah Mada, Yogyakarta 5281, Indonesia; 3 Department of Biomedical Laboratory Science, School of Medicine, Eulji University, Daejon 34824, Korea

**Keywords:** adiponectin, endoplasmic reticulum stress, in vitro culture, parthenogenetic activation, pig, somatic cell nuclear transfer

## Abstract

**Simple Summary:**

Successful attenuation of endoplasmic reticulum (ER) stress signaling has a beneficial outcome in in vitro embryonal improvement. We evaluated the effect of adiponectin during in vitro culture in porcine embryos derived from parthenogenetic activation and somatic cell nuclear transfer (SCNT). We found that 15 and 30 μg/mL adiponectin treatment significantly improved cleavage rates, blastocyst formation rates, and total cell number (TCN) of blastocysts derived from parthenogenetic activation and reduced the expression levels of *XBP1*. In SCNT embryos, the cleavage rate, blastocyst formation rate, and TCN of blastocysts were significantly improved by 15 μg/mL adiponectin treatment compared to the control. In addition, the 15 μg/mL adiponectin treatment reduced the levels of *XBP1* expression and ER stress-related genes, increased expression levels of pluripotency-related genes, and decreased apoptosis-related gene expression. Comprehensively, treatment with 15 μg/mL adiponectin enhanced the in vitro developmental capacity of early-stage SCNT porcine embryos by reducing ER stress and apoptosis.

**Abstract:**

The main factor of embryonic demise is endoplasmic reticulum (ER) stress. Successful attenuation of ER stress results in an improvement in embryo development. We studied the impact of adiponectin in the in vitro culture (IVC) of porcine embryos derived from parthenogenetic activation and somatic cell nuclear transfer (SCNT). The first experiment revealed that 15 and 30 μg/mL adiponectin treatments improved cleavage, blastocyst rates, and total cell number (TCN) of parthenogenetic embryos and reduced the expression of *XBP1* compared to the 5 μg/mL adiponectin treatment and control groups (*p* < 0.05). The second experiment showed that cleavage rate, blastocyst formation rate, and TCN of blastocysts were improved in the 15 μg/mL adiponectin treatment group compared with the control group, with significantly reduced *XBP1* expression in ≥4-cell stage SCNT embryos and blastocysts (*p* < 0.05). Treatment with 15 μg/mL adiponectin significantly improved the expression of *XBP1* and reduced the expression of ER stress-related genes (*uXBP1, sXBP1, PTPN1*, and *ATF4*), increased the expression levels of pluripotency-related genes (*Nanog* and *SOX2*), and decreased apoptosis-related gene expression (*Caspase*-3). These results suggest that 15 μg/mL adiponectin enhanced the in vitro developmental capacity of early-stage SCNT porcine embryos by reducing ER stress and apoptosis.

## 1. Introduction

Adiponectin is a protein hormone, a subset of cytokines that are derived from adipose tissue. Adiponectin affects glucose metabolism, fertility, and inflammation [1]. Furthermore, adiponectin regulates various signaling pathways in organisms, such as mitogen-activated protein kinase (MAPK), serine/threonine protein kinase (AKT), AMP-activated protein kinase (AMPK), peroxisome proliferator-activated receptor α (PPARα), and nuclear factor-κB [2,3,4,5]. Adiponectin is localized in follicular cells, oocytes, corpus luteum, and follicular fluid [6]. Adiponectin receptors have been detected in the cerebrospinal fluid of humans, mice, rats, and in the hypothalamus of humans and pigs [7,8,9]. The adiponectin receptor consists of two isoforms, namely, AdipoR1 and AdipoR2 [1]. The structure of the two receptors is analogous to the topology of G-protein-coupled receptors with intracellular/extracellular orientation of the N-terminus and C-terminus [2]. Adiponectin forms various higher-order structures along with a trimeric form and low and high molecular weight form, all of which have diverse mechanisms in metabolic homeostasis [10]. Levels of adiponectin in the bloodstream vary from 3 to 30 μg/mL in various species [2,11,12,13,14,15,16]. In mice, AdipoQ, AdipoR1, and AdipoR2 are expressed in all stages of preimplantation embryos [17]. AdipoR1 and AdipoR2 are strongly expressed in the preimplantation embryo period, and AdipoR1 and AdipoR2 have been detected in human endometrial epithelial and stromal cells [18]. In a rabbit experiment, AdipoQ, AdipoR1, and AdipoR2 were detected in blastocysts and uteri during the preimplantation period [19,20]. These findings support that adiponectin plays a role in embryo development and implantation.

Recent studies have revealed that adiponectin has important functions, such as steroidogenesis, oxidative stress, and apoptosis, in germ and somatic cells [11]. In females, it functions in oocyte maturation and preimplantation embryo development [1,11]. Supplementation of 30 μg/mL adiponectin during oocyte maturation or embryo culture in in vitro development of parthenogenetically activated embryos improved the maturation and development rates of embryos in pigs [21]. Treatment with 5 μg/mL adiponectin during in vitro maturation (IVM) of goat oocytes improved maturation rates by regulating the MAPK pathway [22]. In mouse experiments, adiponectin reduces endoplasmic reticulum (ER) stress and apoptosis of adipocytes in vivo and in vitro by upregulating the AMPK/PPARα/activating transcription factor-2 (*ATF2*) pathway [23]. Moreover, treatment with adiponectin on bovine mammary epithelial (MAC-T) cells reduced activation of ER stress on the proliferation of MAC-T cells [24]. Therefore, we hypothesize that adiponectin has a strong relationship with ER stress in pigs.

ER is a membranous organelle that is essential for lipid metabolism, calcium metabolism, protein synthesis, folding, and transportation. ER also plays a role in the cellular response to stress [25,26]. In vitro embryo production may suffer various stresses during oocyte collection for embryo culture processes [27]. With respect to embryo development, the successful attenuation of ER stress signaling has a beneficial outcome of embryos on in vitro embryonal development [28]. In porcine in vitro embryos, modulation of the unfolded protein response (UPR) signaling pathways and reduction of ER stress during IVM restored meiotic maturation rates [29]. Moreover, treatment with the ER stress inhibitor tauroursodeoxycholic acid (TUDCA) during late-cleaving embryos improved embryo quality and development [30]. These findings imply that the alleviation of ER stress is important in early-stage embryo development.

In vitro embryo production involves various steps such as ovarian harvesting, oocyte selection, oocyte maturation, oocyte fertilization, parthenogenetic activation (PA), somatic cell nuclear transfer (SCNT), and embryo culture. Due to the low capacity of pig embryo development from the cleavage stage until the blastocyst stage compared to its in vivo counterparts [31], researchers have been attempting to improve these techniques and subsequently improve the in vitro production of embryos [32]. Recent studies on the establishment of chemically defined media, utilization of the ‘zona hardening’ technique [32], and improvement of maturation rates [33,34] have proved successful. Moreover, various treatments to improve embryo development, such as zebularine [35], adiponectin [21], quisinostat [36], suberoylanilide hydroxamic acid [37], LAQ824 [38], melatonin [29], and spermine [39] have been reported. LAQ824 and Quisinostat are potent histone deacetylase inhibitors (HDACi) known to inhibit class I, IIa, and IIb histone deacetylase [36,40]. HDACi treatment has been used to improve embryo development in SCNT and PA embryos [41]. In addition, LAQ824 treatment enhanced SCNT blastocyst rate by upregulating histone 3 lysine 9 (H3K9) and histone 4 lysine 12 (H4K12) levels [38]. Quisinostat treatment could improve SCNT embryo development by upregulating the epigenetic nuclear reprogramming status [36]. Zebularine, a DNA methyltransferase inhibitor, has been reported to alter DNA methyltransferase levels and improve porcine SCNT embryo competence [35]. Adiponectin treatment improved maturation of oocytes and blastocyst rate in porcine PA embryos through the p38MAPK pathway [21]. Suberoylanilide hydroxamic acid has been reported to improve the interspecies SCNT of dog–pig embryos [37]. Melatonin treatment during in vitro maturation improved the meiotic maturation rate and reduced ER stress [29]. Melatonin also supports lipid metabolism and is important for oocyte maturation and embryo development [42]. Spermine supplementation enhanced oocyte maturation, embryo development, and concomitantly increased intracellular glutathione levels and decreased reactive oxygen species levels [39]. However, no previous study has investigated the relationship between adiponectin and ER stress in the early embryonic development of SCNT. Therefore, the purpose of this study was to investigate the relationship between adiponectin treatment and the ER stress signaling pathway and apoptosis in early-stage SCNT porcine embryos.

## 2. Materials and Methods

### 2.1. Ethics Approval and Chemicals

The study was conducted in conformity with the Institutional Animal Care and Use Committee (IACUC) of Seoul National University (approval No. SNU-190621-2). All reagents and chemicals were purchased from Sigma-Aldrich (St. Louis, MO, USA) unless otherwise specified.

### 2.2. Retrieval of Oocyte and IVM

Ovaries were obtained from Landrace prepubertal gilts at a local pig abattoir and placed at 32–37 °C in 0.9% normal saline solution. Oocyte collection and IVM were performed as previously explained [33,35,42,43]. In brief, Oocytes were collected from follicles (3–6 mm) utilizing a 10-mL syringe. Follicular fluid containing cumulus-oocyte complexes COCs was aspirated and then washed at least three times in a porcine washing medium containing 9.5 g/L of tissue culture medium (TCM)-199 (1X) Earle’s salts (Cat. No. 31100-027) (Thermo Fisher Scientific, MA, USA), 10 mM N-piperazine-N′- (2-ethanesufonic acid) (HEPES), 0.3% polyvinyl alcohol (PVA), 2 mM sodium bicarbonate, 5 mM sodium hydroxide, and 1% penicillin-streptomycin (Invitrogen). We selected COCs with a compact multi-layer of cumulus cells and dark, evenly granulated ooplasm for the experiment. Immature oocytes were cultured in IVM medium containing of TCM-199 Earle’s salts (Cat. No. 11150-059), 10% porcine follicular fluid (pFF) (*v/v*), 10 ng/mL epidermal growth factor, 0.57 mmol/L cystein, 0.91 mmol/L sodium pyruvate, 10 IU/mL human chorionic gonadotropin, 10 IU/mL equine chorionic gonadotropin, and 5 mg/mL insulin at 39 °C in a humidified atmosphere of 5% CO_2_ in air for 22 h. Then, COCs were washed, relocated into hormone-free IVM medium, and then cultured for 22 h.

### 2.3. Parthenogenetic Activation

Parthenogenetic activation methods were performed as explained formerly [44]. Briefly, After IVM, denuded oocytes were stabilized in activation medium comprises with 0.28 M mannitol, 0.1 mM CaCl_2_, 0.5 mM HEPES, and 0.1 mM MgSO_4_. Next, oocytes were transferred into an activation chamber. Electric activation of oocytes was performed by using a BTX Electro-Cell Manipulator 2001 (BTX Inc., San Diego, CA, USA) with a single direct current (DC) pulse of 1.5 kV/cm for 60 μs. Oocytes were transferred to porcine zygote medium-5 (PZM-5) (Waco Chemicals, Osaka, Japan, Cat. CSR-CK024) after electrical activation, then cultured at 39 °C in a humidified atmosphere of 5% O_2_, 5% CO_2_, and 90% N_2_.

### 2.4. Cell Isolation, Nuclear Donor Cell Culture, and Arrangement

Porcine fibroblasts were obtained from the ear tissue of an adult pig then cleaned as explained previously [36,45], hairs were removed using a sterile surgical blade and washed four to five times with phosphate-buffered saline (PBS, Gibco, Grand Island, NY, USA). Cell preparation was performed as explained in a previous study [36,46]. The minced tissue was cultured in Dulbecco’s modified Eagle’s medium (DMEM; Gibco, culture medium) consists of 1 mM sodium pyruvate, 10% fetal bovine serum (Gibco, culture medium) (*v/v*), and 100 IU/mL each of penicillin and streptomycin at 38.5 °C, 5% CO_2_ in humidified air. Donors for SCNT were used from cells from passages 3 to 7. The cell suspension was prepared by using trypsin and placed in Tyrode’s albumin lactate pyruvate (TALP)-HEPES for SCNT.

### 2.5. SCNT and Embryo Culture

SCNT processes including enucleation, nuclear transfer, fusion, and activation were performed as explained formerly [38]. In brief, oocytes were denuded with 0.1% hyaluronidase in TALP-HEPES medium and stained with 5 μg/mL Hoechst-33342 in TALP-HEPES for 10 min. Oocytes with dark homogenous ooplasm with polar body were selected for SNCT [33]. Furthermore, the first polar body and elemental Metaphase II (MII) chromosome were enucleated using an aspiration glass pipette. Then, a donor cell was transferred using an aspiration glass pipette into the perivitelline space of enucleated oocyte. The medium for enucleation was TALP medium consisting of 5 μg/mL cytochalasin B (CB) and TALP without CB for nuclear transfer injection medium. The couplets were stabilized into fusion medium (0.28 M mannitol enclosing 0.1 mM MgSO_4_ and 0.5 mM HEPES) and placed in a 20 μL droplet of fusion medium for electrically induced fusion. Hereinafter, oocyte-donor cell couplets fusion was implemented using an electrical machine (LF101; Nepa Gene, Chiba, Japan) with a single DC pulse of 1.2 kV/cm, 30 μs. The couplets were cultured for 1 h in PZM 5 medium, then equilibrated in activation medium (0.28 M mannitol enclosing 0.1 mM CaCl_2_, 0.1 mM MgSO_4_, and 0.5 mM HEPES) and accomplished by electrical stimulation with a single DC pulse of 1.5 kV/cm for 30 μs using a BTX Electro Cell Manipulator 2001 (BTX Inc., San Diego, CA, USA). SCNT embryos were rinsed several times in PZM-5 culture medium, and approximately 15–20 embryos were cultured in 20 μL PZM-5 droplets topped with mineral oil at 38.5 °C in a humidified atmosphere of 5% CO_2_, 90% N_2_, and 5% O_2_.

### 2.6. Experimental Design and Adiponectin Treatment

In the first experiment, we supplemented various concentrations of adiponectin during IVC on parthenogenetic porcine embryos. Experimental design in the first experiment was as follows: (i) adiponectin 0 μg/mL (control); (ii) adiponectin 5 μg/mL; (iii) adiponectin 15 μg/mL; and (iv) adiponectin 30 μg/mL. Adiponectin (Cat No. RD572023100, BioVendor) was supplemented into the culture medium during IVC. In the second experiment, we utilized the 15 μg/mL adiponectin treatment during IVC for SCNT: (i) adiponectin 0 μg/mL (control) and (ii) adiponectin 15 μg/mL. In the first and second experiment, we studied the effects of adiponectin on cleavage rate and following embryo development, total cell numbers (TCN) of blastocysts, and X-box binding protein 1 (*XBP1*) expression levels. The experiment was replicated at least three times in each analysis. In the third experiment, we analyzed the mRNA expression levels of the gene associated with ER stress, apoptosis, and embryo quality from ≥4 cell stage embryo derived from SCNT on day-2. We evaluated two groups: (i) adiponectin 0 μg/mL (control) and (ii) adiponectin 15 μg/mL.

### 2.7. Embryo Development and Total Blastocyst Cells Assessment

The embryo development calculation as day 0 is based on the day of electrical activation for PA and SCNT-derived embryos. Cleavage and blastocyst rates were evaluated on day-2 (48 h) and day-7 (168 h), respectively. Further, the TCN of blastocysts is counted by the nuclei staining technique [43]. Blastocysts were rinsed in TALP solution, immediately stained with 5 μg/mL Hoechst-33342 in a dark environment for 10 min. Blastocysts were placed on glass slides with a tear of glycerol, topped with a cover glass, and evaluated by a fluorescence microscope (TE 2000; Nikon Corp, Tokyo, Japan). Image J software (version 1.49 q; National Institutes of Health, Bethesda, MD, USA) was utilized to analyze the images.

### 2.8. Immunofluorescence Staining of XBP1 in Embryos

Protein levels of *XBP1* were analyzed by immunofluorescence staining described previously [33]. In the first experiment, we performed on blastocyst stage of PA-derived embryos. At least 20 blastocysts each group from four biological replicates were utilized. In the second experiment, we performed on embryos at ≥4 cell stage and at the blastocyst stage. We utilized at least 20 samples of ≥4 cell embryos or blastocysts per group from four biological replicates. In brief, samples were washed with PBS with 1% polyvinyl alcohol (PVA; *w/v*), then sample fixation was implemented for 1 h in 4% paraformaldehyde (*w/v*) in PBS. The samples were percolated with 1% (*v/v*) Triton X-100 in distilled water (DW) at 38 °C incubator for 1 h, and then the embryos were rinsed in 1% PVA in PBS. Further, to prevent nonspecific binding, the samples were incubated in 2% bovine serum albumin (BSA) in PBS for 2 h. Then, embryos were transferred to 2% BSA in PBS consist of *XBP1* primary antibody (1:400; PA5-27650; Invitrogen, Carlsbad, CA, USA), incubated at 4 °C for the night. Next, 1% PVA in PBS solution was used for washing the embryos. Embryos were rinsed in PVA and incubated in a secondary fluorescein isothiocyanate-conjugated anti-rabbit polyclonal antibody (1:200; ab6717; Abcam, Cambridge, UK) for 2 h in room temperature at 25 °C. Dilution of antibody was implemented using 2% BSA in PBS. Further, the embryos were arranged on a glass slide with 100% glycerol and captured with epifluorescence microscope (TE2000-S; Nikon Corp., Tokyo, Japan). Image J software (version 1.46r; National Institute of Health, Bethesda, MD, USA) was used to analyze the fluorescence images.

### 2.9. Analysis of Gene Expression on ≥4 Cell Stage Embryo on Day-2 by Quantitative Real-Time PCR (qRT-PCR)

The samples were collected from at least 400 of ≥4 cell stage embryo from at least 8 biological replicates in each group and preserved at −80 °C until use. RNA extraction, complementary DNA (cDNA) synthesis, and processes associated quantitative real-time PCR (qRT-PCR) were implemented as formerly described [38,47]. We used the RNAqueous^TM^ Micro Kit (Invitrogen, Vilnius, Lithuania). mRNA quantification was implemented utilizing a NanoDrop2000 spectrophotometer (Thermo Fisher Scientific, Wilmington, DE, USA). According to the company’s guideline, cDNA was synthesized immediately using amfiRivert cDNA synthesis Platinum Master Mix 0 (GenDEPOT, Houston, TX, USA). For quantitative real-time PCR, composition of every single reaction is consisting of 10 μL SYBR Premix Ex Taq (Takara, Otsu, Japan), 8.2 μL of Nuclease Free Water, 1 μL cDNA, 0.4 μL (10 pmol/μL) forward primer, and 0.4 μL (10 pmol/mL) reverse primer. These were placed in a 96-well reaction plate (Micro-Amp Optical 96-Well Reaction Plate, Applied Biosystems, Singapore). The StepOne^TM^ Real-Time PCR System (Applied Biosystems, Waltham, MA, USA) was used for amplification of the mixture. Thermal cycler was performed 40 PCR cycles with parameters: initial denaturation at 95 °C for 15 s, annealing at 60 °C for 1 min, and extension at 72 °C for 1 min. we performed at least three replicates for each plate. The mRNA expression levels of each gene were normalized to the housekeeping gene (*GAPDH*). The list of primer sequences is presented in Table 1. Each transcript sample was calculated by using the equation R = 2^−^[ΔCt sample − ΔCt control]. For simplification of correlation, the average expression level of each gene from the control group was set as 1.

### 2.10. Statistical Analysis

The data were evaluated with GraphPad PRISM 5.01 (PRISM 5; GraphPad Software, Inc., San Diego, CA, USA). All data from the first experiment were evaluated by using univariate analysis of variance (ANOVA) followed by Tukey’s test. In addition, all data from the second and third experiments concerning gene expression levels were evaluated by Student’s *t* test. Probability values less than 0.05 (*p* < 0.05) were regarded to be statistically significant.

## 3. Results

### 3.1. Effects of Adiponectin Supplementation on Embryo Development, TCN of Blastocyst, and Expression of XBP1 Derived from Parthenogenetic Activation

**Figure 1:**
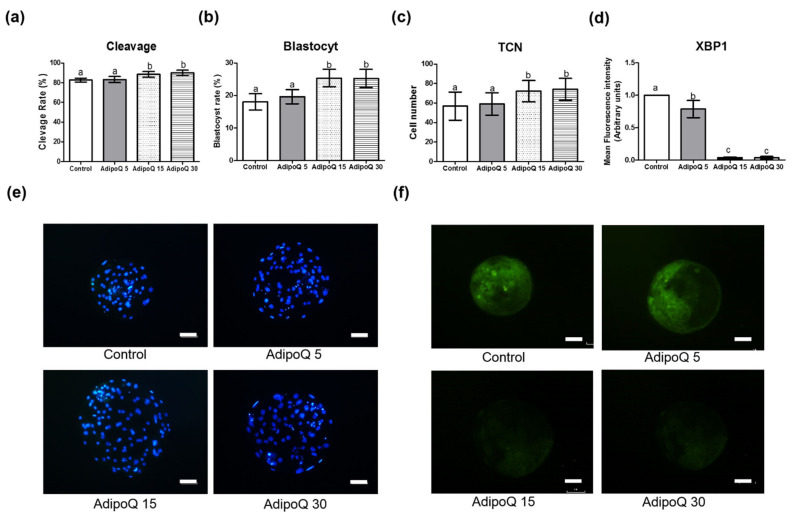
Effect of adiponectin supplementation of various concentrations during in vitro culture of parthenogenetic activation embryos on cleavage rate (**a**), blastocyst formation rate (**b**), total cell numbers (**c**), and protein expression of *XBP1* (**d**); Hoechst staining of blastocysts (Scale bars 50 μm) (**e**) and *XBP1* immunofluorescence (scale bars 50 μm) (**f**). Data are expressed as mean ± SD. a,b Bars with different letters indicate significant difference (*p* < 0.05). The experiment was performed as at least three independent replicates. AdipoQ 5 = adiponectin 5 μg/mL; AdipoQ 15 = adiponectin 15 μg/mL; AdipoQ 30 = adiponectin 30 μg/mL. TCN, total cell number of blastocysts.

In the first experiment, after parthenogenetic activation of oocytes, the effects of adiponectin on cleavage rate, embryo development, TCN of blastocysts, and protein expression of *XBP1* were investigated (Figure 1). We applied various concentrations of adiponectin during IVC for 168 h. Results showed that 15 and 30 μg/mL adiponectin supplementation enhanced the development of cleavage, blastocyst, and TCN of blastocysts compared to the control and 5 μg/mL adiponectin groups (*p* < 0.05). Furthermore, 15 and 30 μg/mL adiponectin treatment reduced the expression levels of *XBP1* compared to the control and 5 μg/mL adiponectin groups (*p* < 0.05). Interestingly, there were no significant differences between 15 and 30 μg/mL adiponectin treatment on cleavage, blastocyst, TCN of the blastocyst, and expression levels of *XBP1*.

### 3.2. Effects of Adiponectin Supplementation during IVC on Embryo Development, TCN of Blastocyst, and Expression of XBP1 Derived from SCNT

**Figure 2:**
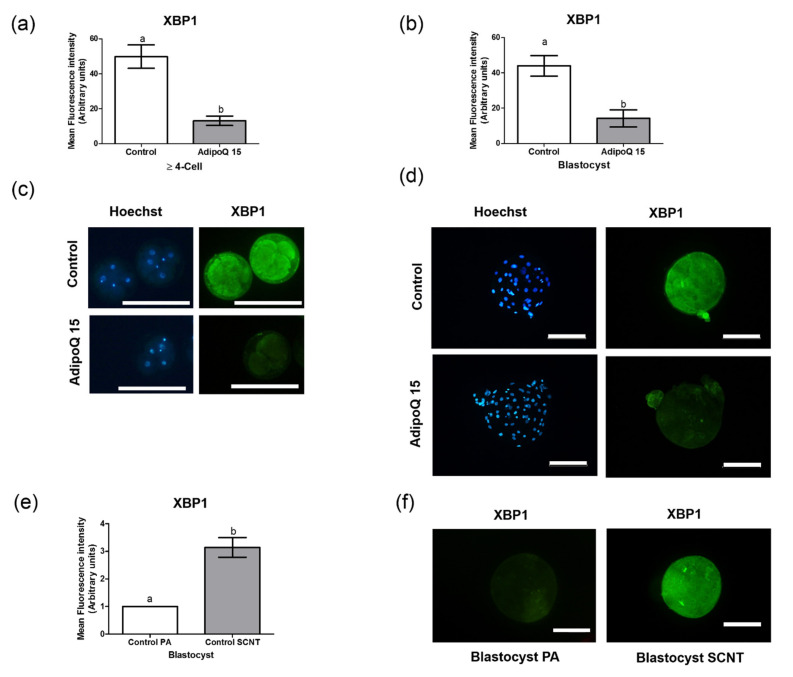
Effect of 15 μg/mL adiponectin supplementation during in vitro culture of somatic cell nuclear transfer (SCNT) embryos on the protein expression of *XBP1* in ≥4-cell embryos on day-2 (**a**) and in blastocysts (**b**); Hoechst (blue) and immunofluorescence (green) staining of control and adiponectin-treated ≥4-cell embryos (scale bars 200 μm) (**c**) and blastocysts (scale bars 100 μm) (**d**). Comparison of *XBP1* expression in the blastocyst stage derived from parthenogenetic activation and SCNT (scale bars 100 μm) (**e**,**f**). At least 24 embryos per group from 4 biological replicates were analyzed in each experiment. Data are expressed as mean ± SD. a,b Values with letters indicate significant difference (*p* < 0.05). The experiment was performed as at least three independent replicates. AdipoQ 15 = adiponectin 15 μg/mL.

In experiment 2, we studied the effects of adiponectin on cleavage, blastocyst rate, TCN of blastocysts, and protein expression of *XBP1* in ≥4-cell embryos and blastocysts by performing the SCNT technique. According to the results of the first experiment, 15 μg/mL adiponectin treatment was utilized in the second experiment. The use of 15 μg/mL adiponectin supplementation yielded a significant enhancement in cleavage, blastocyst formation rate, and TCN compared to the control treatment (*p* < 0.05) (Table 2). In addition, analysis of *XBP1* expression level revealed that 15 μg/mL adiponectin treatment significantly reduced the level of *XBP1* expression in ≥4-cell stage embryos and blastocysts compared to the control treatment (*p* < 0.05) (Figure 2).

### 3.3. Effects of 15 μg/mL Adiponectin Supplementation during IVC on Unfolded Protein Response-Related Genes Caspase-3, Nanog, and SOX2 in ≥4-Cell Day-2 Embryos

**Figure 3:**
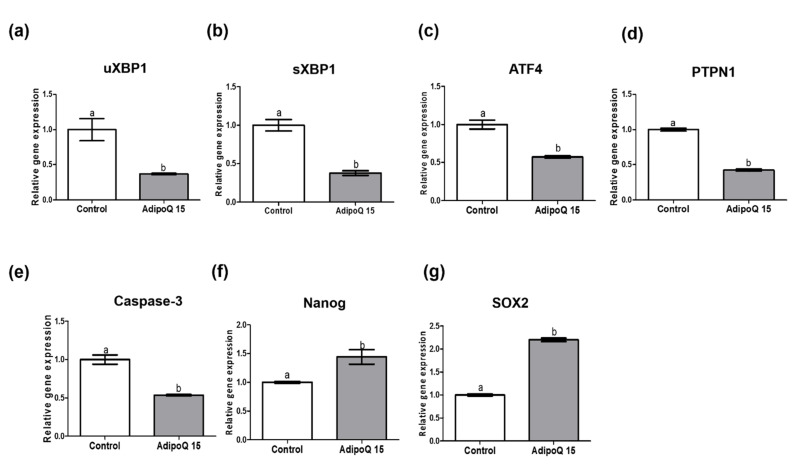
Relative expression levels of unfolded protein response-related mRNAs (*uXBP1*, *sXBP1*, *ATF4*, *PTPN1*), *Caspase*-3, *Nanog, and SOX2* in ≥4-cell embryos (day 2) derived from somatic cell nuclear transfer embryos (**a**–**g**). Data are expressed as mean standard deviation (±SD). Within each category, a,b groups marked with different letters are significantly different (*p* < 0.05). The experiment was replicated three times. AdipoQ 15 = adiponectin 15 μg/mL.

In experiment 3, we analyzed the effects of 15 μg/mL adiponectin treatment on the expression of UPR genes, *Caspase*-3, *Nanog,* and *SOX2* in ≥4-cell day-2 SCNT embryos (Figure 3). Treatment with 15 μg/mL adiponectin significantly reduced the expression levels of *uXBP1*, *sXBP1*, *PTPN1*, *ATF4*, and apoptosis-related gene *Caspase*-3 compared to the control treatment (*p* < 0.05). Moreover, the expression levels of pluripotency-related genes (*Nanog* and *SOX2*) were lower than those in the control (*p* < 0.05).

## 4. Discussion

In vitro embryo production by means of parthenogenetic activation, in vitro fertilization (IVF), and embryo culture techniques could be crucial for agricultural and biomedical purposes, as these biotechnologies could ensure good development and quality of embryos. Along with the success of IVF-IVC technologies, crucial improvements have been achieved in SCNT through the enhancement of enucleation methods, fusion, activation, and production efficiency [48,49]. However, embryos derived from current IVC systems still face the major challenge of low development rate and quality compared to in vivo-derived embryos [50,51]. The major contributor to this failure in in vitro embryonic development is ER stress signaling, and factors that modulate or alleviate ER stress signaling have resulted in positive effects on embryo survival and further development capacity [28]. The reduction of ER stress is progressively performed in in vitro culture treatments in order to increase the efficiency of parthenogenetic activation, SCNT, and IVF. Examples include the use of tauroursodeoxycholic acid [52,53], melatonin [29], valproic acid [54], and salubrinal [55]. In the present study, we studied the supplementation of adiponectin during IVC on embryo development, ER stress, embryo quality, and apoptosis.

In the first experiment, we found that 15 and 30 μg/mL adiponectin supplementation significantly increased cleavage rates, blastocyst rates, and TCN of parthenogenetic embryos. Moreover, there were no significant differences between 15 and 30 μg/mL adiponectin treatment during IVC. Concomitantly, these treatment groups significantly reduced the expression level of *XBP1* compared to the control and 5 μg/mL adiponectin groups (*p* < 0.05). We demonstrated that treatment with 15 and 30 μg/mL adiponectin significantly improved cleavage rate, blastocyst rate, and TCN of blastocysts compared to the control and 5 μg/mL adiponectin groups (*p* < 0.05). These results implied that 15 μg/mL adiponectin treatment during IVC was sufficient to enhance in vitro embryo development. The results were similar between 15 and 30 μg/mL adiponectin treatment, with no significant difference among the groups (*p* > 0.05). In addition, a previous study reported that 30 μg/mL adiponectin improved oocyte maturation rate and blastocyst formation rates in porcine oocytes [21]. Even though 30 μg/mL is the upper limit of physiological concentration [11,56], investigation on higher concentrations of adiponectin treatment could provide further insight regarding the evaluation of the effects of adiponectin on in vitro embryo development.

Adiponectin directly affects the development of preimplantation embryos of mice in vitro [1]. In porcine embryos, adipoR1 and adipoR2 were localized in cumulus cells, oocytes, and early-stage embryos [21]. Furthermore, adiponectin improves the in vitro maturation of oocyte and embryo development through the p38MAPK pathway [21], reduces ER stress-induced early apoptosis, and blocks the mitochondrial apoptosis pathway through the AdipoR1/AMP-activated protein kinase signal pathway in mouse adipose tissue [23]. Treatment by 10 and 20 μg/mL adiponectin in human umbilical vein endothelial cells (HUVECs) for 24 h after stimulation by lipopolysaccharide reduced the number of apoptotic endothelial cells, implying that adiponectin treatment reduced apoptosis in endothelial cells by alleviating the ER stress *IRE1*α pathway stimulated by oxidative stress [57]. Many studies have reported that adiponectin has beneficial effects in alleviating ER stress in cells and embryo development [21,23,24,57]. Adiponectin reduced ER stress and apoptosis of endothelial cells by downregulating *GRP78*, *Caspase*-12 expression, and the *IRE1α* pathway in HUVECs [57]. In addition, activation of *IREα* can promote the C/EBP- homologous protein (CHOP) signaling pathway to induce apoptosis [58,59]. Adiponectin also alleviated UPR signaling by downregulating glucose-regulated protein 78 (*GRP78*), eukaryotic translation initiator factor 2α (*eIF2 α*), protein kinase RNA-like ER kinase (*PERK*), and *IRE1α* (*ATF6α*) expression in MAC-T [24].

*XBP1* is an essential activator regulating gene expression of UPR signaling during ER stress. Under stress, *XBP1* transcription is activated to form spliced-*XBP1* (*sXBP1*) from the unspliced-*XBP1* (*uXBP1*) form. Moreover, *XBP1* is spliced specifically under ER stress; therefore, *XBP1* is generally used as an ER stress marker both in vivo and in vitro [27,60]. In addition, *XBP1* expression was detected in the cytoplasm at the 2- to 8-cell, morula, and blastocyst by fluorescence staining assessment [61]. Inhibition of ER stress by alleviating the expression of active *XBP1* improved embryo development in mice and pigs [29,60,61]. However, day-2 embryos with 5 to 8 cells can be associated with fragmented embryos [62]. Embryos derived from SCNT and parthenogenetic activation revealed a higher occurrence of fragmented embryos whose presence was misinterpreted during observation under a microscope [63,64]. Furthermore, fragmentation may significantly decrease blastocyst rate and TCN of blastocysts in humans and pigs [64,65]. The fragmentation rates at the 5- to 8-cell stage were 28.7% and 40.8% in parthenogenetic activation and SCNT embryos, respectively [62]. In contrast, no fragmentation was observed in porcine embryos derived in vivo, this was associated with microfilament distribution between in vitro and in vivo-derived embryos [64]. The cause of embryonic fragmentation may be related to in vitro culture conditions [65]. Moreover, in vitro culture conditions produce a higher concentration of reactive oxygen species (ROS) that trigger developmental disturbance of the embryos [66]. Many studies have reported the use of chemically defined media such as PVA for IVM supplementation. These reports revealed that PVA supplementation of IVM medium can be utilized for oocyte maturation comparable to pFF-supplemented medium [67,68,69]. Therefore, further investigation of embryo development stages associated with adiponectin, in vitro culture defined media, and ER stress could contribute to the enhancement of in vitro embryo development.

Our present study showed that 15 and 30 μg/mL adiponectin treatments reduced the expression levels of *XBP1* in blastocysts derived from parthenogenetic activation. *XBP1* is one of the classic ER stress marker genes; other ER stress-related genes include *ATF4*, *GRP78*, *HSPA5*, and *ATF6* [27]. In addition, *XBP1* has an important function in the IVM of oocytes and in vitro embryo development [27,61]. The s*XBP1* and *uXBP1* mRNAs were expressed at the 4-cell, morula, and blastocyst stages of porcine embryos, while the *XBP1* protein is expressed in the cytoplasm and nucleus during the 4-cell, morula, and blastocyst stages [60]. In addition, ER stress-activated *XBP1* splicing may play role early embryonic genome activation in porcine [60]. In the second experiment, 15 μg/mL adiponectin treatment during the IVC of SCNT embryos enhanced cleavage, blastocyst rate, and TCN of the blastocysts, and concurrently reduced *XBP1* expression levels in ≥4-cell embryos and blastocysts (*p* < 0.05). A comparison of *XBP1* expression between blastocysts derived from parthenogenetic activation and SCNT revealed that SCNT embryos showed significantly higher expression than parthenogenetic activation embryos. In addition, SCNT embryos received more manipulation than parthenogenetic activation embryos, including enucleation of first polar body, MII chromosome mass, and nuclear transfer of somatic cells [38]. In the present study, adiponectin improved PA and SCNT embryo development in the early stage and concomitantly decreased *XBP1* expression. Investigation of ER stress in porcine SCNT and IVF embryos has been previously reported. Lee et al. reported that SCNT embryos showed increased expression levels of *XBP1* compared with IVF embryos in all stages of preimplantation embryos [70]. Therefore, manipulation of oocytes in SCNT embryos can induce excessive ER stress and apoptosis during preimplantation development compared to PA and IVF embryos. Investigation of *XBP1* during embryo development has been reported in previous studies, and *XBP1* protein expression was detected in germinal vesicles, MII, 1-, 2-, 4-, 8-cell, morula, and blastocyst stages of porcine PA embryos. In addition, expression of *XBP1* was low during the MII, 1-, 2-, and 8-cell stages, but high during the morula and blastocyst stages [60]. Furthermore, mRNA expression of *XBP1* was strong during the 1-cell stage and weak during the blastocyst stage in porcine SCNT and IVF embryos [70]. In contrast, ER stress conditions were observed during the 1-cell stage but were strong in the blastocyst stage of mouse embryo development [71].

In the third experiment, we evaluated the gene expression related to *UPR (uXBP1*, *sXBP1*, *PTPN1*, *and ATF4*), apoptosis (*Caspase*-3), and embryo development (*Nanog* and *SOX2*) in SCNT 4-cell embryos. During in vitro embryo production processes, including parthenogenetic activation and SCNT, the oocytes and embryos face various stresses [27]. Stress on the ER initiates the unfolded protein response mechanism. ER stress initiates the segregation of *GRP78/BiP* from three branches of transmembrane proteins (*ATF6*, *IRE1α*, and *PERK*) [27,33]. *ATF6*, *IRE1α*, and *PERK* actively correct protein folding, enact ER homeostasis, and eliminate misfolded proteins [72]. Activation of PERK promotes translation of *ATF4*, inducing transcription of genes such as *GRP78* or *BiP* [73]. Furthermore, *IRE1*α is activated and triggers the conversion of *uXBP1* to *sXBP1*, which subsequently stimulates UPR-reactive genes [27]. ER stress also promotes *PTPN1* gene expression, which is associated with ER stress. Therefore, the reduction of *XBP1* alleviates ER stress levels [74].

Our present study revealed that 15 μg/mL adiponectin treatment significantly reduced the mRNA expression of ER stress-associated genes (*uXBP1*, *sXBP1*, *PTPN1*, and *ATF4*) in early-stage SCNT embryos. These results demonstrated that 15 μg/mL adiponectin treatment alleviated the ER stress-associated genes. Under ER stress conditions triggered by tunicamycin, expression of *u*-*XBP1* and *s*-*XBP1* in the 4-cell, morula, and blastocyst stages increased [60]. Moreover, tunicamycin treatment during IVM in porcine embryos increased the expression of ER stress-related genes, including *u*-*XBP1* and *s*-*XBP1* in oocytes [33]. In this study, 15 μg/mL adiponectin treatment reduced expression of *u*-*XBP1* and *s*-*XBP1* to the same degree at the transcriptional level. In addition, adiponectin treatment also reduced the expression levels of other genes related to ER stress such as *ATF4* and *PTPN1.* Based on these results, adiponectin treatment reduced ER stress by downregulating UPR-related genes such as *uXBP1*, *sXBP1*, *PTPN1*, and *ATF4*. A previous study reported a relationship between *XBP1* and adiponectin. *XBP1* overexpression triggered inflammation of adipocytes by downregulating adiponectin and activating IL-6, TNF-, and leptin expression [75]. A study on mitochondrial dysfunction and ER stress revealed that treatment with S-methylisothiourea sulfate significantly reduced *sXBP1* expression and promoted adiponectin synthesis in adipocytes [76]. However, further investigation is needed to elucidate the relationship between adiponectin, ER stress-related genes, and the improvement of in vitro embryo production.

Analysis of the apoptosis-related gene *Caspase*-3 revealed that it was significantly downregulated in 15 μg/mL adiponectin treatment compared to the control (*p* < 0.05). Similar results were obtained with TUDCA treatment during the IVC of porcine embryos. The presence of TUDCA increased the TCN of blastocysts and increased anti-apoptotic gene expression (*BCL2*), however, the pro-apoptotic genes *BCL2L1* (*Bcl*-*xl*) and *TP53* were downregulated [60]. In addition, adiponectin has a positive effect on the reduction of apoptosis; the number of apoptotic endothelial cells caused by sepsis was attenuated following treatment with adiponectin. Furthermore, adiponectin reduced apoptosis of endothelial cells by alleviating ER stress through the inositol-requiring enzyme 1α (*IRE1*α) pathway [57]. At the protein level, adiponectin treatment increased *PPARα* expression, reduced *ATF2* protein levels, and reduced apoptosis in mouse adipocytes [23]. Treatment with adiponectin in HUVECs reduced ER stress and apoptosis by decreasing *p*-*IRE1αI*, *GRP78*, *CHOP*, and *Caspase*-12 protein levels [57]. Moreover, treatment with adiponectin in rat ventricular myocytes alleviated ER stress response by reducing the levels of *GRP78*, *Caspase*-12, C/EBP homologous protein, and p38MAPK [77]. However, additional studies are needed to investigate the relationship between adiponectin and ER stress, particularly on the protein levels of key genes related to ER stress and mechanisms of adiponectin associated with ER stress in specific stages of porcine in vitro embryo development.

In addition, mRNA expression levels of genes related to embryo development (*Nanog* and *SOX2*) were high in the 15 μg/mL adiponectin treatment group. *Nanog* is expressed in the inner cell mass (ICM) and functions to maintain pluripotency. In addition, *SOX2* is related to the pluripotent embryonic stem cells (ESCs) phenotype [38]. Therefore, apoptosis is related to blastocyst quality [38]. Accordingly, the results of 15 μg/mL adiponectin treatment provide constructive evidence supporting ESCs. Moreover, the cleavage rate, blastocyst formation rate, and total cell number of blastocysts significantly improved in the 15 μg/mL adiponectin treatment due to firm regulation of apoptotic genes.

## 5. Conclusions

The present study revealed that 15 μg/mL adiponectin treatment in IVC enhanced the developmental capacity of early-stage porcine SCNT-derived embryos by reducing the level of *XBP1* expression and alleviating ER stress-related genes. In addition, 15 μg/mL adiponectin treatment enhanced the TCN of blastocysts and the developmental capacity of early-stage SCNT embryos by enhancing the expression levels of *Nanog* and *SOX2* and decreasing that of *Caspase*-3.

## Figures and Tables

**Table 1:** Primer sequences for qRT-PCR.

Genes	Primer Sequences (5′–3′)	Product Size (Bp)	Accession No.
*GAPDH*	F: GTCGGTTGTGGATCTGACCT R: TTGACGAAGTGGTCGTTGAG	207	NM_001206359
*uXBP1*	F: CATGGATTCTGACGGTGTTG R: GTCTGGGGAAGGACATCTGA	106	NM_001142836.1
*sXBP1*	F: GGAGTTAAGACAGCGCTTGG R: GAGATGTTCTGGAGGGGTGA	142	NM_001271738.1
*ATF4*	F: AGTCCTTTTCTGCGAGTGGG R: CTGCTGCCTCTAATACGCCA	80	NM_001123078.1
*PTPN1/* *PTP1B*	F: GGTGCTCACGACTCTTCCTC R: TTCTCTGCACGAGCTTCTGA	158	NM_001113435.1
*Caspase*-3	F: GCCATGGTGAAGAAGGAAAA R: GGCAGGCCTGAATTATGAAA	132	NM_214131.1
*Nanog*	F: GGTTTATGGGCCTGAAGAAA R: GATCCATGGAGGAAGGAAGA	98	NM_001129971
*SOX2*	F: ATGCACAACTCGGAGATCAG R: TATAATCCGGGTGCTCCTTC	130	NM_001123197

qRT-PCR, Quantitative real-time polymerase chain reaction; F, Forward primer; R, Reverse Primer.

**Table 2:** Effect of adiponectin treatment during in vitro culture (IVC) on embryonic development and total blastocyst cell number derived from somatic cell nuclear transfer (SCNT) embryos.

Adiponectin (μg/mL)	Number of Embryos Cultured	No. of Embryos Developed (Mean ± SD, %)		Total Blastocyst Cell Number (Mean ± SEM)
		≥2 Cells	Blastocyst	
0	132	117 (88.6 ± 0.89) ^a^	34 (25.6 ± 3.1) ^a^	55.96 ± 5.5 ^a^
15	130	120 (92.3 ± 0.55) ^b^	53 (39.8 ± 3.1) ^b^	72.44 ± 7.0 ^b^

Note: Replication number = 5. Abbreviation: SD: standard deviation. ^a,b^ Values within a column with different superscripts differ significantly at *p* < 0.05.

## Data Availability

Information supporting the results of this investigation will, upon suitable request, be accessible from the corresponding author.
